# Laparoscopic resection of aortocaval paraganglioma diagnosed by serial increase in urinary metanephrines after bilateral adrenalectomy in a patient with multiple endocrine neoplasia type 2A

**DOI:** 10.1002/iju5.12345

**Published:** 2021-07-04

**Authors:** Yushi Miyata, Koji Hatano, Yosuke Okuno, Takeshi Ujike, Shinichiro Fukuhara, Motohide Uemura, Hiroshi Kiuchi, Ryoichi Imamura, Michio Otsuki, Norio Nonomura

**Affiliations:** ^1^ Department of Urology Osaka University Graduate School of Medicine Suita Japan; ^2^ Department of Metabolic Medicine Osaka University Graduate School of Medicine Suita Japan

**Keywords:** laparoscopic surgery, multiple endocrine neoplasia type 2, paraganglioma, pheochromocytoma, urinary metanephrine

## Abstract

**Introduction:**

Although bilateral pheochromocytoma is prevalent in patients with multiple endocrine neoplasia type 2, extra‐adrenal tumors rarely occur in the aortocaval area.

**Case presentation:**

A 35‐year‐old man with multiple endocrine neoplasia type 2A (*RET* codon Cys634Arg mutation) underwent bilateral adrenalectomy for metachronous pheochromocytoma. After bilateral adrenalectomy, urinary metanephrines decreased below the measurement sensitivity. The levels of urinary metanephrines were serially elevating to a peak of 187 ng/mgCr during the 11‐year follow‐up period; however, urinary normetanephrine levels remained almost stable. ^123^I‐metaiodobenzylguanidine single‐photon emission computed tomography revealed abnormal accumulation with a mass of 25 × 18 mm in diameter in the aortocaval space cranially to the renal vessels. The extra‐adrenal paraganglioma was successfully resected using transperitoneal laparoscopic surgery.

**Conclusion:**

The serial increase in urinary metanephrine levels was useful for the detection of the recurrent tumor in a patient who had undergone bilateral adrenalectomy.

**Abbreviations**
^123^I‐MIBG SPECT: ^123^I‐metaiodobenzylguanidine single‐photon emission computed tomography
CT: computed tomography
MEN2: multiple endocrine neoplasia type 2
MN: metanephrine
NMN: normetanephrine

**Keynote message**

A serial increase in MNs by spot urine samples can be a useful marker for the early diagnosis of recurrent tumors in patients with MEN2 after bilateral adrenalectomy, leading to the detection of tumor localization by ^123^I‐MIBG SPECT.

## Introduction

MEN2 is an autosomal dominant genetic disorder caused by mutations in the *RET* gene. Although the prognosis of MEN2 depends mainly on medullary thyroid cancer aggressiveness, pheochromocytoma is a major component of the syndrome, known to have an increased incidence with age.^1^ Bilateral pheochromocytoma is prevalent, whereas extra‐adrenal paraganglioma has been considered rare.^2^, ^3^, ^4^, ^5^, ^6^ In 2014, Castinetti *et␣al*. reported that extra‐adrenal tumors occurred in 5 out of 563 patients with pheochromocytomas associated with MEN2. All the tumors were in the aortocaval space close to the adrenal glands, in five patients who already had bilateral pheochromocytomas.^7^ Although Osinga *et␣al*. reported that the urinary metanephrine (MN) concentration after bilateral adrenalectomy was lower than that in the reference population,^8^ the reference value of urinary MNs for the detection of recurrent tumors has not yet been established. Here, a case of an extra‐adrenal paraganglioma that was diagnosed with a serial increase in urinary MNs after bilateral adrenalectomy in a patient with MEN2A has been described.

## Case presentation

**Fig. 1:**
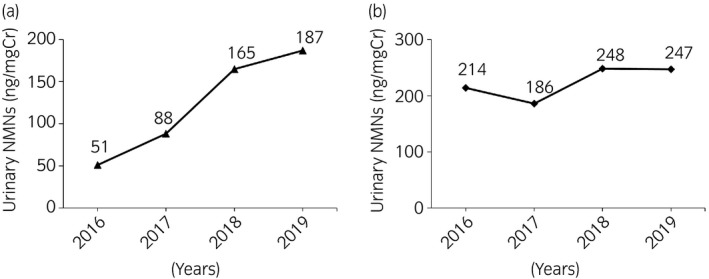
The levels of (a) urinary metanephrine (MN) and (b) urinary normetanephrine (NMN) during follow‐up.

**Fig. 2:**
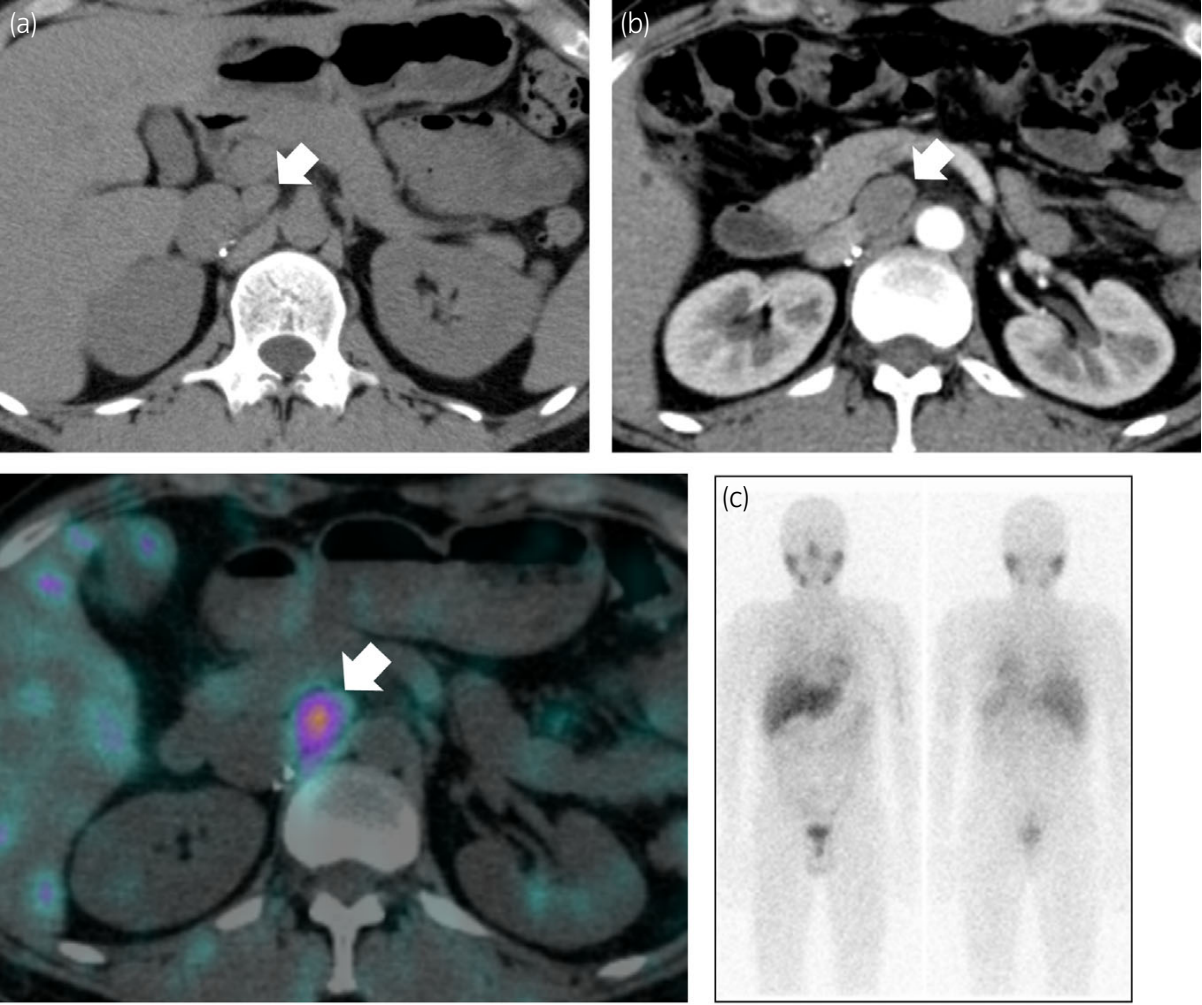
CT showing a tumor in the aortocaval area cranially to the renal vessels. The tumor size increased during follow‐up: (a) 14 × 9 mm in diameter in 2017 and (b) 25 × 18 mm in diameter in 2019. (c) ^123^I‐MIBG SPECT (left) and scintigraphy (right) in 2019. Arrows: tumor.

A 35‐year‐old man underwent left laparoscopic partial adrenalectomy in 2003 and right laparoscopic transperitoneal adrenalectomy in 2008, with metachronous bilateral pheochromocytoma. After bilateral adrenalectomy, urinary MN levels decreased to below the measurement sensitivity. In 2010, the patient underwent total thyroidectomy for medullary thyroid carcinoma. After surgery, hydrocortisone and levothyroxine were administrated, without any other history of drug administration. He was diagnosed with MEN2A (*RET* codon Cys634Arg mutation) by genetic testing. In 2016, he was referred to our hospital because of elevated urinary MNs measured by spot urine samples. The levels of urinary MNs were serially elevated from 51 to 187 ng/mg Cr between 2016 and 2019 with no apparent symptoms (Fig 1a). The levels of urinary NMN remained almost unchanged (Fig␣1b). In 2017, CT showed a mass of 14 × 9 mm in diameter in the aortocaval area cranially to the renal vessels (Fig 2a). In 2019, CT revealed that the aortocaval mass grew to 25 × 18 mm in diameter (Fig.␣2b). ^123^I‐MIBG SPECT revealed abnormal accumulation of the mass (Fig.␣2c). A decision was made to perform a laparoscopic resection of the tumor based on the diagnosis of an extra‐adrenal paraganglioma. Doxazosin was administered, and the dose was gradually increased to 16 mg.

**Fig. 3:**
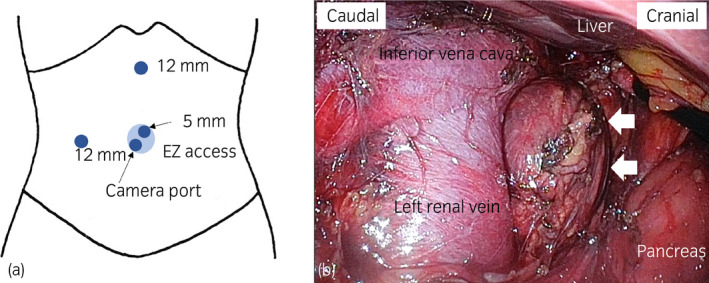
Port placement and intraoperative findings during tumor resection. (a) After a small paraumbilical incision, a LAP PROTECTOR and EZ access (Hakko Medical, Nagano, Japan) were placed into the abdominal cavity, and two additional incisions were made in the abdominal wall for placement of the laparoscopic ports. (b) Tumor found in the aortocaval space superior to the left renal vein. Arrows: tumor.

The patient was placed in the left lateral decubitus position. A small paraumbilical incision and two additional incisions were made to place the transperitoneal laparoscopic ports (Fig 3a). After mobilization of the ascending colon and duodenum, the tumor was located in the aortocaval space superior to the left renal vein (Fig 3b). The tumor was completely resected, without severe adhesions. The intraoperative blood pressure transiently increased to 165/67 mmHg, but this was not sustained. The operation time was 121 min, and the blood loss was 20 mL. No perioperative complications occurred.

Pathological findings of the tumors are shown in Figure␣4. The aortocaval tumor existed adjacent to the ganglion, with positive immunohistochemical staining for synaptophysin and chromogranin A. The tumor showed a Ki‐67 labeling index of 5%. No ectopic adrenal tissue was observed around the tumor. After surgery, urine MN decreased to 17 ng/mg Cr and remained stable. No recurrence was observed 15 months postoperatively.

**Fig. 4:**
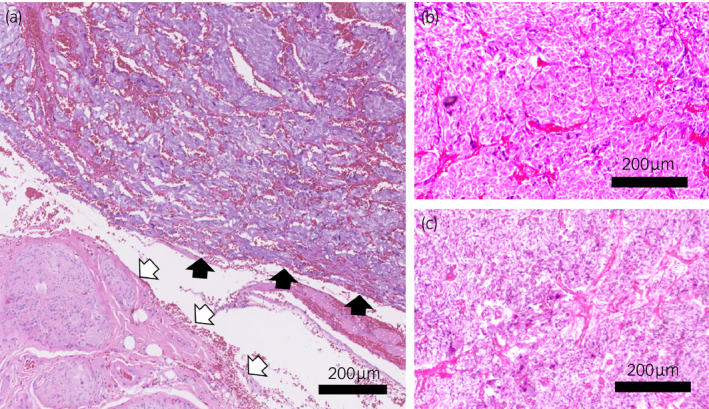
Pathological findings of the tumors by hematoxylin and eosin stain. (a) The aortocaval paraganglioma existed adjacent to the ganglion, with a GAPP score of 4 and a PASS score of 2. There was no lymph node tissue and no ectopic adrenal tissue surrounding the tumor. Black arrows: tumor. White arrows: ganglion. (b) Left pheochromocytoma resected in 2003, with a PASS score of 0. (c) Right pheochromocytoma resected in 2008, with a PASS score of 0.

## Discussion

Although the adrenal medulla and sympathetic nervous system represent the sources of catecholamine production in healthy individuals, the single largest source of MNs is the adrenal medulla.^9^ Consequently, bilateral adrenalectomy is expected to be followed by a decrease in urinary MN concentrations. Osinga *et␣al*. reported that the median urinary MN concentration 3 to 6 months after bilateral adrenalectomy was lower than that in the reference population (7 [1–22] μmol/mol creatinine vs. 61 [49–74] μmol/mol creatinine; p<0.05), and the changes in urinary MNs persisted during follow‐up.^8^ In contrast, urinary NMN concentrations increase after bilateral adrenalectomy.^8^ Thus, the use of incorrect reference intervals interferes with the early detection of tumor recurrence when the associated rise in urinary MN concentration occurs within the reference range applicable to healthy individuals.^8^ Amar *et␣al*. reported that recurrent pheochromocytoma could be detected before the onset of symptoms, based on an increase in urinary MN plus NMN.^10^ In von Hippel–Lindau disease, the tumor diameter of a contralateral pheochromocytoma was correlated with urinary MN and NMN concentrations.^10^ In our case, the tumor size of an extra‐adrenal paraganglioma in MEN2A was correlated with the levels of urinary MNs, but not those of NMNs. The plasma‐free MNs may also be available for the detection of recurrent pheochromocytoma.^11^

Extra‐adrenal paraganglioma in MEN2 can rarely occur, but predominantly in the aortocaval space close to the adrenal glands.^7^ The laparoscopic excision of retroperitoneal paraganglioma is challenging because of demanding localization, and extensive experience is required.^12^ Paragangliomas located cranially to the renal vessels can be safely resected using either laparoscopic or retroperitoneoscopic surgery.^13^, ^14^ Surgeons should be familiar with detaching maneuvers around great vessels and the mobilization of adjacent organs, and careful preoperative planning is mandatory.^14^ In properly selected patients, transumbilical single‐port laparoscopic surgery can be used for retroperitoneal paragangliomas.^15^ Here, transumbilical plus two‐port laparoscopic surgery was selected for safety because of the challenging nature of the aortocaval paraganglioma.

In the present case, the patient had a mutation in *RET* codon 634. Imai *et␣al*. reported that most patients with a *RET* codon 634 mutation develop pheochromocytomas as well as medullary thyroid carcinoma during their lifetimes.^16^ A *RET* codon 634 mutation is associated with a risk of aggressive medullary thyroid carcinoma.^17^ Although the association between codon‐specific *RET* mutations and the potential risk of extra‐adrenal paraganglioma is unknown, the aggressive nature of codon 634 requires careful follow‐up.

## Conclusion

An aortocaval paraganglioma was successfully resected using transperitoneal laparoscopic surgery. A serial increase in urinary MN levels can be a useful marker for the early detection of recurrent tumors in patients who have undergone bilateral adrenalectomy.

## Conflict of interest

The authors declare no conflict of interest.

## Approval of the research protocol by an institutional review board

Not applicable.

## Informed consent

All human subjects provided written informed consent with guarantees of confidentiality.

## Registry and the registration no. of the study/trial

Not applicable.
